# Muscle hyperplasia and hypertrophy dynamics in rainbow trout (*Oncorhynchus mykiss*): impact of high plant-based protein and additive mixtures on muscle physiology

**DOI:** 10.1017/S0007114525105175

**Published:** 2025-10-28

**Authors:** Krishna Pada Singha, Michael Phelps, Ken Overturf, Vikas Kumar

**Affiliations:** 1 Aquaculture Research Institute, Department of Animal, Veterinary & Food Sciences, University of Idaho, Moscow, ID 83844-2160, USA; 2 Department of Animal Sciences, Washington State University, Pullman, WA 99164-6310, USA; 3 USDA-Agricultural Research Service, Hagerman Fish Culture Experiment Station, Hagerman, ID 83332, USA; 4 Department of Biological Sciences, Bowling Green State University, Bowling Green, OH 43403, USA

**Keywords:** Fishmeal replacement, Muscle fibre, Fibre recruitment, Myogenic gene, Myostatin

## Abstract

The effects of high plant-based proteins (PP) used as alternative protein sources in aquafeeds on muscle cellularity and myogenic factors of rainbow trout, *Oncorhynchus mykiss*, remain unclear. This study explored muscle fibre growth phases and the impact of two additive mixtures (A) in high-PP diets on muscle physiology. Over a seven-month trial, 2000 fish (2·22 g) were divided into four groups (five replicates each) and fed isonitrogenous (fry, 46 %; fingerling, 44 %; and grow-out, 42 % crude protein) and isolipidic (20 % lipid) diets: control (30 % fishmeal), PP, PP + A1 (krill meal, taurine, selenium) and PP + A2 (proline, hydroxyproline, vitamin C). Sampling for muscle histology and myogenic gene expression was conducted at ten sampling points from Day 0 to Day 214. Muscle histology (fibre distribution: small, 0–20 μm; small-medium, 20–60 μm; large-medium, 60–100 μm and large, ≥ 100 μm diameter) revealed four growth phases: hyperplasia (2·2–15 g), hypertrophy (15–50 g), hyperplasia (50–150 g) and hypertrophy (150–350 g). MyoD2 and myogenic regulatory factor 4 (MRF4) were upregulated during hyperplasia, while myostatin 1 (MSTN1)/myostatin 2 (2) and reduced Paired box 7 indicating growth inhibition and fewer satellite cells. The PP diet without additives altered fibre recruitment, while PP + A2 enhanced hypertrophy, increasing large (> 100 μm) fibres. Additive mixtures modulated myogenic gene expression, with PP + A2 promoting MyoD2, myogenin and MRF4 and reducing MEF2A/C, contrary to known hypertrophy markers. PP + A1 and PP + A2 diets reduced MSTN1 expression, potentially mitigating growth inhibition. Additive supplementation in PP diets alleviates negative impacts on muscle cellularity and myogenic regulation. The identified growth phases provide insights for precision nutrition, supporting improved feeding strategies for sustainable aquaculture.

Skeletal muscle is a dynamic tissue that undergoes remarkable growth and remodelling throughout the life cycle of a fish^(1)^. Constituting 40–60 % of the total body mass^(2–4)^, the growth and development of skeletal muscle are critical factors influencing the productivity and economic value of many commercially important fish species, including rainbow trout. Unlike mammals, in which muscle fibre recruitment ceases, and post-natal growth depends on hypertrophy^(5)^, the growth of skeletal muscle in fish comprises both mechanisms: hyperplasia, which involves an increase in the number of muscle fibres, and hypertrophy, which is an increase in the size of existing muscle fibres^(1,6)^. The relative contributions of hyperplasia and hypertrophy to muscle growth vary considerably among fish species^(7)^, developmental stages^(8)^, muscle types^(9)^, feeding regimes^(10)^ and various other environmental factors^(11,12)^. Fish growth at different life stages based on the muscle fibre recruitment pattern could be divided into three distinct stages, i.e. embryonic myogenesis, stratified hyperplasia and mosaic hyperplasia^(6)^. In fish, lifelong muscle fibre recruitment is especially important for commercial aquaculture species such as rainbow trout. Among the three distinct growth phases, mosaic hyperplasia – characterised by the presence of fibres with varying diameters in a mosaic-like pattern, occurring from the late larval to adult stages^(6)^ – is particularly important from a commercial aquaculture perspective. This phase is the longest, requiring a substantial amount of feed for the fish to grow to marketable size.

Muscle hyperplasia and hypertrophy processes are regulated by a network of myogenic regulatory factors (MRF). These MRF, including MyoD, myogenic factor 5 (Myf5), myogenin and MRF4, contain a highly conserved DNA-binding domain and play crucial roles in regulating the proliferation, differentiation and fusion of myogenic progenitor cells, also referred to as satellite cells^(1,4,13,14)^. The primary MRF, MyoD and Myf5, direct the commitment of proliferating satellite cells towards the myogenic lineage, while the secondary MRF, myogenin and MRF4, along with myocyte enhancer factor 2 (MEF2) family (i.e. MEF2A and MEF2C), control the subsequent differentiation and fusion of myoblasts into multinucleated myofibres^(15)^. Apart from MRF, other factors, such as myostatin (MSTN), a potent negative regulator of muscle growth^(16)^, have been implicated in modulating the balance between hyperplasia and hypertrophy in fish^(15)^. Moreover, the paired box 7 (Pax7) gene is a marker for muscle satellite cells and is essential for muscle development during growth and repair after injury in fish^(17)^.

Plant-based proteins can modulate muscle growth, cellularity and texture in fish when used to partially or completely replace fishmeal. Higher inclusions of plant proteins tend to reduce white muscle fibre diameter and cross-sectional area, indicative of impaired hypertrophic growth^(18,19)^. This reduced muscle growth is often associated with the downregulation of MRF like MyoD, myogenin, MRF4 and muscle structural genes like myosin heavy chains^(19,20)^. Moreover, the expression of growth-related genes like insulin-like growth factors, mechanistic target of rapamycin and MSTN can also be affected by plant-based diets^(21)^. However, these negative impacts could be alleviated through supplementation with feed additives, which promote growth by enhancing muscle cellularity and positively regulating myogenic factors. Additives such as krill meal, taurine and organic Se have shown promising effects on promoting muscle growth and improving muscle fibre properties in salmonids and other various fish species^(22–26)^. Additionally, proline, hydroxyproline and vitamin C have also demonstrated beneficial effects on muscle growth and collagen synthesis in various fish species^(27–30)^. Most studies have focused on using single additives to enhance fish performance, but from an industrial perspective, combining multiple additives in aquafeeds offers significant advantages. Feed manufacturers frequently incorporate additive mixtures to achieve synergistic effects, optimise formulations and improve production efficiency. The duration of feeding trials is a key factor in assessing the impact of high plant-based diets on muscle growth. While short-term studies have shown that complete fishmeal substitution does not negatively affect muscle growth^(31–33)^, long-term feeding without fishmeal has been linked to reduced growth performance in rainbow trout^(34,35)^.

This study aimed to investigate the contributions of hyperplasia and hypertrophy in muscle growth, as well as the molecular control exerted by the myogenic regulatory factors in the skeletal muscle of rainbow trout during different growth stages through a long-term feeding trial. It was hypothesised that plant-based diets could impair muscle cellularity and its regulatory factors, while additive mixtures might counteract these effects. While our previous studies explored the effects of supplementing high plant–protein diets with these additive mixtures on growth, nutrient utilisation^(36)^ and fillet quality^(37)^, demonstrating improvements in growth performance, nutrient utilisation, fatty acid composition, textural profile and collagen content, the current study focuses on the underlying muscle physiology and the temporal effects of myogenic regulatory gene expression. The research highlighted the synergistic potential of combining additives and the importance of extended feeding trials to assess their long-term efficacy. Additive mixture 1 included krill meal, taurine and organic Se, while additive mixture 2 comprised proline, hydroxyproline and vitamin C, aligning with industrial practices to enhance the practical relevance of the findings for aquaculture. This is the first study investigating how muscle hyperplasia and hypertrophy occur in rainbow trout with regard to dietary effects on muscle physiology and temporal effects of myogenic regulatory gene expression.

## Experimental methods

### Experimental fish

The Riverence Holdings LLC supplied the rainbow trout eggs (fertilised) to the Cold-Water Lab at the Aquaculture Research Institute (ARI), University of Idaho (Moscow, ID), where the eggs were hatched and reared until the desired size (approximately 2·0 g) for the feeding trial.

### Formulation and preparation of experimental diets

Four experimental diets were formulated (Table 1) to satisfy the nutrient requirements of three growth stages (fry, fingerlings and grow-out) of rainbow trout based on NRC (2011)^(38)^. The crude protein (CP) levels of the diets for fry, fingerlings and grow-out stages were 46 %, 44 % and 42 %, respectively, with a 20 % lipid level for all growth stages. The experimental diets were fishmeal-based diet, plant protein-based diet (PP), PP-based diet supplemented with additive mixture 1 of Antarctic krill meal, taurine and organic selenium (PP + A1) and PP-based diet supplemented with additive mixture 2 of L-proline, L-hydroxyproline and vitamin C (PP + A2). Additive dosages were selected based on previous literature. In PP + A1, krill meal was included at 5 %, as trout showed comparable growth to the fishmeal-fed diet at 4·5 %^(39)^, while taurine was added at 0·5 %, the recommended requirement for rainbow trout^(40,41)^. Organic selenium at 3 mg/kg showed the highest growth and tissue Se levels in rainbow trout^(42)^. In PP + A2, L-proline and L-hydroxyproline were included based on their roles in boosting muscle collagen at 0·75 % in turbot^(43)^ and improving growth and feed efficiency at levels up to 0·5 % L-hydroxyproline in Chu’s croaker^(44)^. Vitamin C was added at supranutritional levels based on its role in promoting growth and collagen synthesis in Atlantic salmon^(45)^ and its synergistic effect with proline^(46)^.

**Table 1.** Feed formulation and proximate composition for different experimental diets

		Experimental diets^†^											
		46 % CP				44 % CP				42 % CP			
Ingredients (%)		FM	PP	PP + A1	PP + A2	FM	PP	PP + A1	PP + A2	FM	PP	PP + A1	PP + A2
Fishmeal^‡^		30	10	10	10	30	10	10	10	30	10	10	10
Soybean meal^§^		8	12	12	12	8	12	12	12	8	12	12	12
Canola meal^||^		6·25	8	8	8	6·25	8	8	8	6·25	8	8	8
Soy protein concentrate^§^		12·52	23·76	19·37	21·6	12·05	21	16·61	18·85	12	20	15·62	17·85
Corn protein concentrate^¶^		7	10	10	10	5·65	9·5	9·5	9·5	4·1	8·35	8·35	8·35
Wheat gluten meal^††^		7	10	10	10	5·6	9·5	9·5	9·5	4·06	8·3	8·3	8·3
Fish oil^‡‡^		15·59	17·89	17·17	17·9	15·62	17·9	17·18	17·91	15·66	17·92	17·21	17·94
Wheat flour^§§^		10·74	5·45	5·06	6·05	13·93	9·2	8·81	9·79	17·03	12·53	12·12	13·11
Dicalcium phosphate^||||^		1·2	1·2	1·2	1·2	1·2	1·2	1·2	1·2	1·2	1·2	1·2	1·2
Choline chloride^||||^		0·6	0·6	0·6	0·6	0·6	0·6	0·6	0·6	0·6	0·6	0·6	0·6
Vitamin premix ARS 702^¶¶^		0·8	0·8	0·8	0·8	0·8	0·8	0·8	0·8	0·8	0·8	0·8	0·8
Trace mineral premix ARS 1440^***^,^*^		0·1	0·1	0·1	0·1	0·1	0·1	0·1	0·1	0·1	0·1	0·1	0·1
Stay-C 35^†††^,^**^		0·2	0·2	0·2	0·2	0·2	0·2	0·2	0·2	0·2	0·2	0·2	0·2
Additive Mixture 1	Krill meal^‡‡‡^	0	0	5	0	0	0	5	0	0	0	5	0
	Taurine^§§§^	0	0	0·5	0	0	0	0·5	0	0	0	0·5	0
	Organic Se^||||||^,^*^	0	0	0·0003	0	0	0	0·0003	0	0	0	0·0003	0
Additive Mixture 2	Proline^¶¶¶^	0	0	0	0·75	0	0	0	0·75	0	0	0	0·75
	Hydroxyproline^****^	0	0	0	0·5	0	0	0	0·5	0	0	0	0·5
	Vitamin C^†††^,^**^	0	0	0	0·3	0	0	0	0·3	0	0	0	0·3
Total		100	100	100	100	100	100	100	100	100	100	100	100
Proximate composition (%)													
Moisture		7·94	8·05	7·83	7·56	8·24	7·66	8	8	7·88	8·36	7·96	8·09
Crude protein		45·84	46·15	46·56	46·31	43·71	44·73	45	44	42·32	41·59	41·73	42·44
Crude lipids		18·24	19·19	19·29	19·48	18·6	19·3	19·56	19·54	19·56	18·96	19·7	19·33
Crude fibre		2·17	2·94	2·83	2·95	2·17	2·85	2·74	2·81	1·9	2·57	2·66	2·88
Nitrogen free extract		17·32	17·74	17·56	17·86	18·7	19·65	18·9	19·83	20·68	21·66	21·99	22·29
Total ash		8·49	5·93	5·93	5·84	8·58	5·81	5·8	5·82	7·66	6·86	5·96	4·97

† Experimental diets: FM, Fishmeal-based diet; PP, Plant-based diet; PP + A1, Plant–based diet supplemented with additive mixture 1 (i.e. krill meal, taurine, and organic Se); PP + A2, Plant–based diet supplemented with additive mixture 2 (i.e. L-proline, L-hydroxyproline, and vitamin C).

‡ Menhaden fishmeal, Omega Protein (Houston, TX, USA).

§ Archer Daniels Midland (Decatur, IL, USA).

|| Rangen Inc., (Buhl, ID, USA).

¶ Empyreal® 75, Cargill Corn Milling (Blair, NE, USA).

†† Cargill^TM^ (Germany).

‡‡ Menhaden fish oil, Omega Protein (Reedville, VA, USA).

§§ Pastry flour sifted, Montana Milling (Great Falls, MT, USA).

|||| Alliance Nutrition, ADM affiliate (Twin Falls, ID, USA).

¶¶ Contributed per kg of diet: vitamin A (as retinol palmitate), 30 000 IU; vitamin D3, 2160 IU; vitamin E (as DL-α-tocopheryl-acetate), 1590 IU; niacin, 990 mg; calcium pantothenate, 480 mg; riboflavin, 240 mg; thiamin mononitrate, 150 mg; pyridoxine hydrochloride, 135 mg; menadione sodium bisulfate, 75 mg; folacin, 39 mg; biotin, 3 mg; vitamin B12, 90 μg.

*** Contributed in mg per kg of diet: zinc, 37; manganese, 10; iodine, 5; copper, 3; selenium, 0·4.

††† Ascorbyl polyphosphate Rovomix Stay-C 35 (DSM Nutritional Products Ltd., Basel, Switzerland).

‡‡‡ Antacrtic krill (*Euphausia superba*) meal, Florida Aqua Farms, Inc (Dade City, FL, USA).

§§§ Feed grade taurine (#W381306), Sigma-Aldrich (Burlington, MA, USA)

|||||| Sel-Plex® 3000, Alltech (Nicholasville, KY, USA).

¶¶¶ L-proline, Ajinomoto Co., Inc. (USA).

**** Trans-4-Hydroxy-L-proline (#AC121780250), Thermo Scientific Chemicals (USA).

* All experimental diets were supplied with recommended levels of inorganic Se using ‘Trace mineral premix ARS 1440’; whereas only PP + A1 diet were supplemented with 3 mg/kg of Sel-Plex® 3000 as organic Se.

** All experimental diets were supplied with recommended levels of vitamin C using Stay-C 35, whereas only the PP + A2 diet was supplemented with an additional level of 0·3 % Stay-C 35.

Cold pelleted diets using a laboratory pellet mill (California Pellet Mill Company, San Francisco) fitted with a 1·5- and 2·5-mm die for fry and fingerlings, respectively, were made at the Hagerman Fish Culture Experiment Station (HFCES), University of Idaho. For the grow-out stage, floating pellets (4·5 mm) were prepared using a twin-screw cooking extruder (DNDL- 44, Buhler AG, Uzwil) with an 18-s exposure in six extruder barrel sections at the USDA-ARS facility in Bozeman, MT. All the diets were shipped to the Cold-water lab, ARI, and stored at room temperature until used.

### Proximate and amino acid composition of experimental diets

The proximate composition, including moisture, crude protein, crude lipids, crude fibre and total ash of diets, was measured based on AOAC Official Methods 934·01, 990·03 (combustion analyses, LECO), 920·39 (A), 978·10 and 942·05, respectively^(47)^. The amino acid composition was analysed based on the AOAC Official Methods 982·30 E (a,b,c) chapter 45·3·05^(47)^. Briefly, finely ground samples (200 mg) were used for amino acid composition analysis. Each sample, including two independent replicates, was hydrolysed for 16 h at 110°C in 6·0 N HCl; then the amino acids were separated on a Beckman 6300 Amino Acid Analyzer (Beckman Instruments, Fullerton) equipped with a high-performance, cation exchange resin column. The nitrogen-free extract of diets was calculated as nitrogen-free extract (%) = 100 – (Moisture + Crude protein + Crude lipids + Crude fibre + Total ash). All the values of proximate composition and amino acid composition (only for grow-out diet with 42 % CP) are provided in Table 1 and Table 2, respectively.

**Table 2.** Amino acid composition of experimental diets^*^ (% of dry diet, crude protein 42 %) of the grow-out stage

Amino acids	FM	PP	PP + A1	PP + A2	Requirement^†^
Indispensable amino acids					
Arg	3·01	2·78	2·75	2·71	1·40–1·80
His	1·29	1·27	1·26	1·25	0·50–0·60
Ile	2·47	2·32	2·35	2·24	0·40–1·70
Leu	4·31	4·42	4·46	4·30	1·10–3·40
Lys	3·54	2·72	2·68	2·57	1·30–2·70
Met	1·25	0·98	0·99	0·94	0·40–0·90
Phe	2·35	2·46	2·48	2·40	0·70
Thr	1·97	1·76	1·76	1·71	1·10
Trp	0·70	0·59	0·65	0·54	0·10–0·30
Val	2·70	2·51	2·52	2·42	0·80–1·60
Dispensable amino acids					
Ala	2·61	2·44	2·43	2·36	
Asp	4·61	4·18	4·12	4·01	
Cys	0·74	0·80	0·80	0·77	
Glu	9·43	10·42	10·33	10·29	
Gly	2·09	1·95	1·92	1·88	
Pro	2·53	3·08	3·09	3·69	
Ser	1·82	1·84	1·83	1·83	
Tyr	1·83	1·79	1·86	1·75	
Others					
Taurine	0·16	0·12	0·60	0·17	
Hydroxyproline	008	0·08	0·08	0·45	

* Experimental diets: FM, Fishmeal-based diet; PP, Plant-based diet; PP + A1, Plant–based diet supplemented with additive mixture 1 (i.e. krill meal, taurine, and organic Se); PP + A2, Plant–based diet supplemented with additive mixture 2 (i.e. L-proline L-hydroxyproline, and vitamin C).

† Dietary indispensable amino acids requirement (% of diet) of rainbow trout according to NRC (2011)^(37)^.

### Experimental design and feeding trial

A total of 2000 fish (initial weight 2·2 g) were randomly stocked at 100 fish/tank following a completely randomised design into twenty circular tanks of 60 L volume recirculating aquaculture system. When the fish reached an average weight of 15 g (based on the fishmeal diet group), they were transferred to another recirculating aquaculture system with large tanks (400 L volume). The fish were fed four diets, each with five replications of tanks.

**Figure 1.**
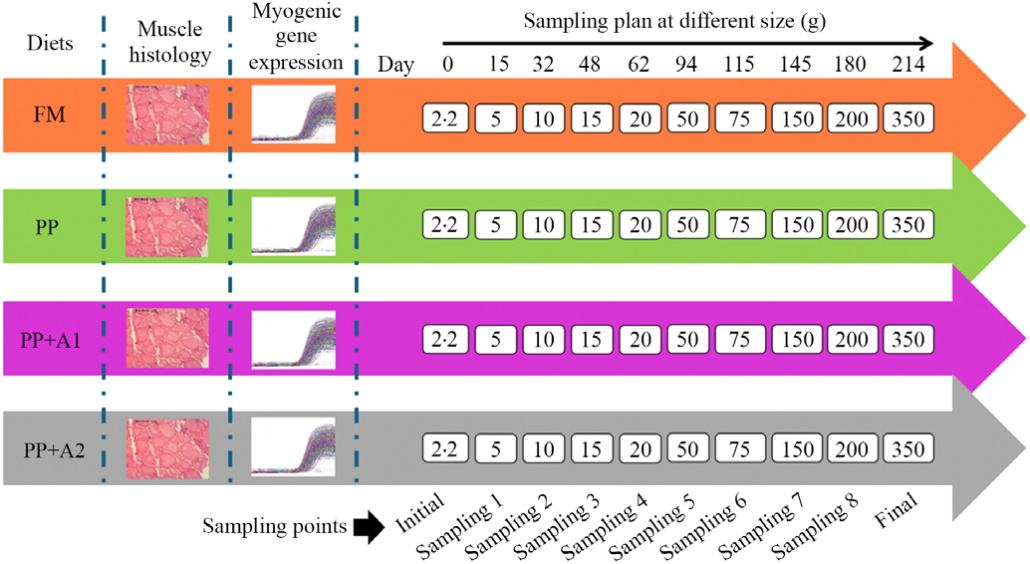
Collection of muscle tissue samples from rainbow trout for histology and gene expression at different sampling points during the seven-month feeding trial.

The water temperature was maintained at 15·0 ± 2°C with a photoperiod of 12 h dark/12 h light. A well-maintained aeration was provided to keep the dissolved oxygen level between 5·0 and 6·0 mg/l. Other water quality parameters such as pH, ammonia-N and nitrate-N were maintained at optimum levels (7·42–7·64, < 0·01 mg/l and 0·1 mg/l, respectively). All water quality parameters during the feeding trial were determined using a commercial kit, Freshwater Aquaculture Kit AQ-2 (#3633–05, LaMotte, Chestertown, MD, USA). The water quality parameters during each specific sampling point (Days) are provided in online Supplementary Table S1. During the 30-week (7-month) feeding trial, fish were fed twice daily to apparent satiation 6 days a week. Fish were fed diets containing 46 % CP until Sampling 2 (Day 32), then 44 % CP until Sampling 4 (Day 62) (Figure 1). After that, they were given experimental diets containing 42 % CP.

### Sampling and analysis protocol

There were ten sampling points, including initial sampling, eight interval samplings (Sampling 1 to 8), and, in the end, final sampling, as depicted in Figure 1. The final weight and length of the fish at each sampling point are provided in online Supplementary Table S2. During initial sampling, ten fish were sacrificed to collect the samples for muscle histology and myogenic gene expression. During the interval and final sampling, three fish from each tank (3 fish × 5 replications = 15 fish for each diet at each sampling point) were euthanised using MS-222 (300 mg/l, buffered to pH 7·0) to collect the samples for muscle histology and myogenic gene expression as described in the following sections.

### Muscle histology

#### Muscle tissue block preparation

The muscle tissue blocks that can be fitted to ‘15 mm × 15 mm × 5 mm’ cryomold (#25 608–924, Tissue-Tek^®^ Cryomold^®^, Sakura^®^ Finetek USA, Inc.) were cut from the anterior portion of the dorsal fin origin from randomly collected fish. Initially, a drop of optimum cutting temperature compound (#25608-930, Tissue-Tek® O.C.T. Compound, Sakura® Finetek USA, Inc.) was put on the cryomold to fix the muscle tissue block in the well of the cryomold which was then dipped into the isopentane (IUPAC name, 2-methylbutane) (#AC126470010, Thermo Scientific™ Chemicals) solution (freezing point, −160·0℃) submerged in liquid nitrogen (N_2_). After some time (20–30 sec), the frozen muscle tissue block with the cryomold was taken out of the isopentane solution, and then an extra optimum cutting temperature compound was added to cover the entire muscle tissue block in the cryomold well. Again, the cryomold was dipped inside the isopentane solution to freeze it and then wrapped with aluminium foil before being dipped in the liquid N_2_ overnight. The isopentane solution was used instead of directly freezing the muscle tissue blocks in liquid N_2_ to avoid boiling liquid N_2_ when in sudden contact with the relatively warm muscle tissue block, which can result in irregular freezing of muscle tissue blocks. The glass beaker containing isopentane solution, when submerged in the liquid N_2_, provides uniform freezing of muscle tissue block and maintains cellular integrity. Finally, the cryomolds with the frozen muscle tissue blocks were stored in a −80℃ freezer from the liquid N_2_ until used for tissue sectioning.

#### Muscle tissue slide preparation

The frozen muscle tissue blocks fixed in the optimum cutting temperature compound were used for cryosectioning at −20 to −22℃ using a cryostat unit (Leica CM1950, Leica Biosystems Nussloch GmbH). Two to three 15–20 μm thin cryosections were then fixed on the single-end frosted glass slides (VWR^®^ Premium VistaVision™, VWR International, LLC) and kept for air drying at room temperature for 5 min. Afterwards, the slides containing muscle tissue sections were used for hematoxylin and eosin stains. Briefly, the slides were first dipped into the hematoxylin solution (#95057-858, VWR^®^ Harris Hematoxylin, VWR International, LLC) for 2 min and then gently washed with deionised water. After air drying at room temperature, the slides were dipped into eosin solution (#95057-484, VWR^®^ Eosin, VWR International, LLC) for 20 sec, and after a gentle wash with deionised water, the slides were kept from air drying at room temperature. Finally, the slides were mounted with rectangular micro cover glasses (#48393-059, VWR® Micro Cover Glasses, VWR International, LLC) using slide mounting media (#14-390-5, Epredia™ Aqua-Mount, Thermo Fisher Scientific Inc.) and kept overnight for permanent mounting.

#### Microscopic imaging and histological analysis

The microscopic images of the slides at 10× magnification were taken using a microscope (Model Eclipse E200LED MV R, Nikon Corporation, Japan). The images were saved with a 100 μm scale bar using the Leica Application Suite X software (Version 3·7·4·23 463, Leica Biosystems Nussloch GmbH, Germany). Two best images from each tank (i.e. 2 × 5 replication = 10 images for each dietary treatment at each sampling point) were selected for image analysis using an image processing software, Fiji^(48)^, to determine the muscle fibre density (i.e. number of muscle fibres per mm^2^ area of the image at 10× magnification) and muscle fibre distribution (i.e. number of different diameter muscle fibres as small, 0–20 μm; small-medium, 20–60 μm; large-medium, 60–100 μm and large, ≥ 100 μm diameter per mm^2^ area of the image at 10× magnification). All the images were analysed manually. The values of muscle fibre density and muscle fibre distribution were averaged for each tank to get five replications for each dietary treatment for statistical analysis.

### Myogenic gene expression

#### RNA extraction from muscle tissue

The muscle tissue from just below the origin of the dorsal fin (after collecting the muscle tissue block sample for histology) was collected. The muscle tissues were stored in 1 ml screwcap cryotubes using RNA stabilisation solution (#AM7021, Invitrogen^TM^ RNA*later*™ solution, Thermo Scientific™, USA) until RNA extraction. For each tank (replication), approximately 100 mg of two muscle tissue samples (ten samples for initial sampling) were put separately in screwcap cryotubes (#16 466–058, VWR® Micro Centrifuge Tube) containing 1 ml of RNA isolation reagent (#TR118, TRI Reagent®, Molecular Research Center, Inc.) and zirconium oxide beads (#E-3396, 1·0 mm diameter, Next Advance, Inc.) used for total RNA extraction following standard protocol. Briefly, muscle tissues were homogenised using a bead homogeniser (Bead Ruptor 96, OMNI International, USA), then 200 μl of chloroform (#MK-4440–1, Macron Fine Chemicals™) was added and shaken for 15 sec. After 3-min incubation at room temperature, the tubes were centrifuged at 12 000 × g for 15 min using a refrigerator centrifuge machine. Afterwards, the aqueous layer from the top of the tubes was carefully transferred to 1·5 ml Eppendorf tubes containing 500 μl of isopropyl alcohol (#67-63-0, Aqua Solutions, Inc.) and mixed gently. After 10 min of incubation at room temperature, the tubes were centrifuged at 12 000 × g for 10 min at 4℃. The supernatant was discarded, and the pellet at the bottom of the tube was washed using 75 % ethanol (#64-17-5, Fisher Bioreagents, Thermo Fisher Scientific Inc., USA) by vortexing and centrifugation at 7500 × g for 5 min thrice. Finally, after removing all the ethanol residue using a micropipette, the pellets were dried at room temperature for 10 min and dissolved using nuclease-free water. The concentration and purity (260/280) of RNA were checked using a Nano-Drop spectrophotometer (NanoDrop One, Thermo Scientific™, USA). The relative quantity and integrity of RNA were confirmed by electrophoresis.

#### cDNA synthesis

Reverse transcription was carried out using the commercial cDNA synthesis Kit (#E3010L, LunaScript® RT SuperMix Kit, New England Biolabs®, Inc.) following the manufacturer’s protocol. Briefly, for each reaction containing a total volume of 20 μl, 4 μl of the LunaScript RT SuperMix (5X), variable volume of RNA samples equivalent to 1 μg of RNA and nuclease-free water to make the volume to 20 μl were added to PCR tubes. The reaction program for cDNA synthesis was set as primer annealing at 25℃ for 2 min, cDNA synthesis at 55℃ for 10 min, and finally, heat inactivation of reverse transcriptase at 95℃ for 1 min. The product was stored at –80°C until the quantitative RT-PCR (qRT-PCR) was performed.

#### Real-Time quantitative PCR

The primers for the housekeeping gene, EF1*α*, and different myogenic genes used are mentioned in Table 3. All the primers were purchased from the Integrated DNA Technologies, USA. For MyoD2, Myf5 and MEF2A genes, normal real-time quantitative PCR in a Real-Time Thermal Cyclers (qTOWER^3^ G, Analytik Jena USA) using the PerfecCTa® SYBR® Green FastMix® (#95 072, Quantabio) was carried out; however, for other myogenic genes (i.e. MEF2C, MyoG, MRF4, MSTN1, MSTN2 and Pax7), a high-throughput (HT) real-time quantitative PCR system (Wafergen SmartChip Real-time PCR system, Takara Bio USA, Inc.) based on SmartChip MyDesign Kit using SmartChip® TB Green® Gene Expression Master Mix (#640211, Takara Bio USA, Inc.) was used. In both cases, the quantification of CT (threshold cycle) of the housekeeping gene was done separately for both systems. Moreover, in both cases, the same real-time quantitative PCR program (as the activation at 95°C for 2 min, then 40 cycle reactions, each of which includes denaturation at 95°C for 10 s, annealing at 61·6°C for 15 s, and extension at 72°C for 10 s) was used for uniformity. Finally, melting curve analysis was done using a temperature gradient to confirm the amplification of the target gene. The relative expression of different myogenic genes was calculated by the 2^–ΔΔCT^ method^(49)^ using the following equation:

**Table 3.** Primers used for growth-related muscle gene expression of rainbow trout fed four experimental diets for seven months

Gene	Abbreviation		Primer sequences	GenBank accession
Elongation factor 1 alpha	EF1α	Forward	5’-TGCCCCTGGACACAGAGATT-3’	NM_001124339.1
		Reverse	5’-CCCACACCACCAGCAACAA-3’	
Myoblast determination protein 1 homolog 2	MyoD2	Forward	5’-GCCGTCACCGACCAACT-3’	Z46924
		Reverse	5’-CACTGTGTTCATAGCACTTGGTAGA-3’	
Myogenic factor 5	Myf5	Forward	5’-CACAAGCTATGGCAACAACTACAG-3’	AY751283
		Reverse	5’- GGCACCAGCACC TCTCT-3’	
Myogenin	MyoG	Forward	5’-CATGGACCGGCGGAAAG-3’	Z46912
		Reverse	5’-GGCCTCGAATGCCTCGT-3’	
Myogenic regulatory factor 4	MRF4	Forward	5’-CACCATGCGTCCAATAAGGAGT-3’	EF450079
		Reverse	5’-AGGCGAAGAAGGCTGGTG-3’	
Myocyte enhancer factor 2A	MEF2A	Forward	5’-GGCCAGGCAGCTCTCA-3’	CA374878
		Reverse	5’-TTGGAGCCCTGAGGTAGGT-3’	
Myocyte enhancer factor 2C	MEF2C	Forward	5’-CCCTAGGCAACCACAACCT-3’	CA380324
		Reverse	5’-ACTGGGAGGTCTATGTGTGACA-3’	
Myostatin 1	MSTN1	Forward	5’-CCGCCTTCACATATGCCAA-3’	NM_001124282.1
		Reverse	5’-CAGAACCTGCGTCAGATGCA-3’	
Myostatin 2	MSTN2	Forward	5’-AGTCCGCCTTCACGCAAA-3’	NM_001124283.2
		Reverse	5’-ACCGAAAGCAACCATAAAACTCA-3’	
Paired box 7	Pax7	Forward	5’-GCCTTTGAGAGGACACACTAC-3’	XM_036947909.1; NM_001258336.1
		Reverse	5’-CCCTTCTGTTGCTGAACCA-3’	

ΔCT = CT value of target gene – CT value of housekeeping gene

ΔΔCT = ΔCT of dietary treatment group* – ΔCT of control group**

*FM, PP, PP + A1 and PP + A2 dietary treatments at different sampling points

**Initial sampling values of each specific gene

### Statistical analysis

For histological measurements (e.g. muscle fibre density and distribution), values were calculated as the number of fibres per mm^2^ area at 10× magnification, representing proportion-based measurements rather than raw counts. Two representative images per tank were analysed and averaged to yield a single continuous data point per tank (*n* 5 per dietary treatment) for statistical analysis. The normality and homogeneity of variance were assessed by examining the Residual *v*. Fitted Plot, Q-Q Residual Plot and Bartlett test. ANOVA was performed using R-programming (R version 4.3.2; RStudio 2023.12.1 + 402) to analyse all the data for significant variation at a 5 % probability level (*P*< 0·05), followed by Tukey’s pairwise comparison with compare the mean values (*n* 5). Based on the balanced study design (four diets with five replicates each), post hoc comparisons were performed using the ‘emmeans’ package in R. The reported standard error of the estimated marginal means (sem) was calculated as

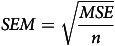




where *MSE* is the residual mean square from the ANOVA, and *n* 5 is the number of replicates per diet. As the sem is identical across all groups, it is reported separately in the table to avoid redundancy.

## Results

### Muscle histology

#### Muscle fibre density and recruitment pattern in control

**Figure 2.**
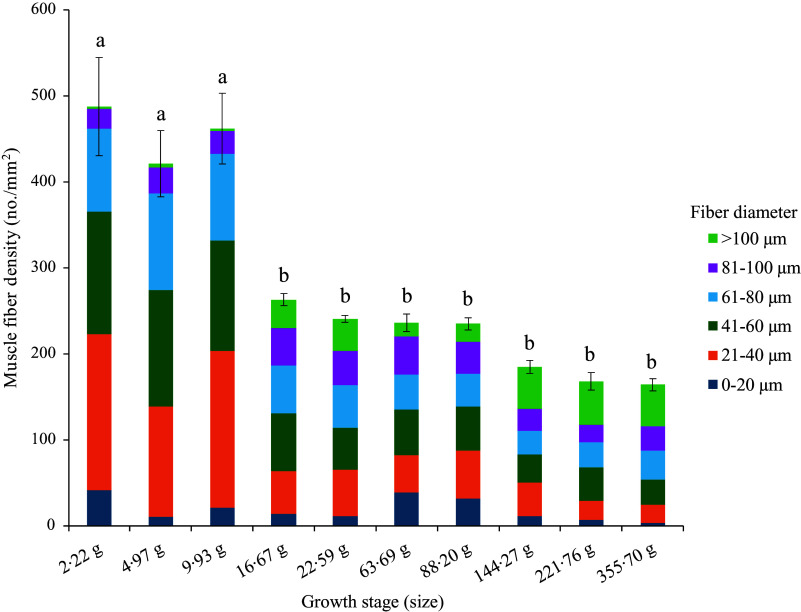
Muscle fibre density and recruitment pattern of rainbow trout based on the control (fishmeal-based) diet fed for seven months.

The muscle fibre density of the control group (FM) significantly reduced at Day 48 (Sampling 3), and thereafter, no significant changes occurred during the rest of the feeding period (Figure 2, Table 4). However, the recruitment of fibres of different sizes showed a complex pattern (Table 4). The recruitment of 0–20 µm diameter fibres was significantly (*P*< 0·05) higher at Day 0 (Initial) and Day 94 (Sampling 5) as compared with Day 214 (Final). However, the recruitment of fibres ranging from 21 to 80 µm diameter followed a similar trend as muscle fibre density. The recruitment of 81–100 µm diameter fibres showed no significant (*P*> 0·05) changes throughout the feeding trial. Interestingly, the fibres with a diameter > 100 µm were found to be significantly higher at Day 48–62 (Sampling 3–4) and Day 145–214 (Sampling 7-Final) as compared with Day 0–32 (Initial-Sampling 2) and Day 94–115 (Sampling 5–6).

**Table 4.** Changes in the recruitment of different-sized fibres^*^ throughout the feeding trial and dietary effect^†^ on the fibre recruitment pattern in rainbow trout muscle

Fibre diameter^‡^	Diets^§^	Initial	Sampling 1	Sampling 2	Sampling 3	Sampling 4	Sampling 5	Sampling 6	Sampling 7	Sampling 8	Final		
		Day 0	Day 15	Day 32	Day 48	Day 62	Day 94	Day 115	Day 145	Day 180	Day 214	sem	*P* value^*^
Fish weight (g)		**2·22**	**4·97**	**9·93**	**16·67**	**22·59**	**63·69**	**88·20**	**144·27**	**221·76**	**355·70**		
0–20 µm	FM	40·95^a^	10·46^ab^	21·09^ab^	13·73^ab^	11·66^ab^	38·44^a^	32·01^ab^	11·44^ab,X^	6·76^ab^	3·38^b^	2·767	**0·003**
	PP	40·95^a^	4·16^b^	21·65^ab^	2·12^b^	15·48^ab^	27·48^ab^	28·89^ab^	6·97^b,XY^	3·23^b^	2·13^b^	2·739	**0·001**
	PP + A1	40·95^a^	18·18^a^	39·49^a^	16·00^a^	9·92^a^	29·30^a^	26·80^a^	2·59^a,Y^	3·60^a^	3·96^a^	3·048	**0·006**
	PP + A2	40·95^a^	19·77^a^	22·10^a^	8·37^a^	8·60^a^	24·09^a^	26·06^a^	1·15^a,Y^	0·47^a^	3·21^a^	3·091	**0·038**
	sem	0	4·27	4·652	2·138	2·303	2·125	1·167	1·359	1·219	0·815		
	*P* value^†^	–	0·576	0·457	0·086	0·769	0·087	0·285	**0·017**	0·363	0·900		
21–40 µm	FM	181·48^a^	128·12^ab^	182·46^a,X^	49·73^c^	53·74^bc^	43·31^c^	55·77^bc^	38·68^c^	21·88^c^	21·35^c^	9·690	**< 0·001**
	PP	181·48^a^	119·48^ab^	98·33^bc,XY^	71·70^bcd^	78·23^bcd^	49·32^bcd^	41·71^cd^	32·39^cd^	29·55^cd^	17·42^d^	8·136	**< 0·001**
	PP + A1	181·48^a^	174·34^a^	89·65^ab,Y^	54·15^b^	60·30^b^	32·76^b^	46·14^b^	30·32^b^	37·08^b^	22·83^b^	9·955	**< 0·001**
	PP + A2	181·48^a^	120·98^ab^	110·26^abc,XY^	74·17^bcd^	56·49^bcd^	35·74^bcd^	34·05^cd^	19·88^d^	14·81^d^	18·85^d^	9·075	**< 0·001**
	sem	0	16·467	13·391	4·625	5·031	3·484	3·217	2·539	3·875	2·146		
	*P* value^†^	–	0·635	**0·041**	0·145	0·325	0·346	0·102	0·052	0·205	0·836		
41–60 µm	FM	143·21^a^	135·78^a^	128·30^ab^	66·94^bc^	48·53^c^	53·69^c^	51·06^c^	33·23^c^	38·89^c^	28·63^c^	7·232	**< 0·001**
	PP	143·21^a^	134·51^a^	99·20^ab^	102·06^ab^	61·80^bc^	63·15^bc^	52·26^bc^	51·48^bc^	49·16^bc^	28·52^c^	6·439	**< 0·001**
	PP + A1	143·21^b^	202·28^a^	119·98^bc^	80·76^cd^	62·04^d^	43·14^d^	47·00^d^	26·72^d^	36·42^d^	29·90^d^	8·578	**< 0·001**
	PP + A2	143·21^a^	131·80^ab^	72·22^cd^	82·82^bc^	66·42^cd^	52·05^cd^	33·35^cd^	37·56^cd^	24·55^d^	31·52^cd^	6·602	**< 0·001**
	sem	0	12·891	8·445	4·802	5·216	3·871	3·362	3·951	3·369	2·421		
	*P* value^†^	–	0·148	0·072	0·063	0·682	0·360	0·170	0·147	0·065	0·974		
61–80 µm	FM	95·85^ab^	112·54^a^	100·51^ab^	55·95^bc^	49·98^c^	40·87^c^	37·78^c^	27·24^c^	29·56^c^	34·09^c^	5·155	**< 0·001**
	PP	95·85^a^	74·48^ab^	77·39^ab^	54·55^abd^	61·20^abc^	41·78^bc^	43·11^bc^	44·11^bc^	37·35^bc^	23·32^c^	3·887	**< 0·001**
	PP + A1	95·85^a^	92·76^a^	87·93^a^	54·59^ab^	42·56^b^	35·61^b^	42·17^b^	26·51^b^	37·42^b^	27·77^b^	4·51	**< 0·001**
	PP + A2	95·85^ab^	99·54^a^	76·03^abc^	55·68^bcd^	46·59^cd^	53·76^bcd^	34·61^cd^	24·84^d^	30·55^d^	29·03^d^	4·545	**< 0·001**
	sem	0	7·582	6·433	3·134	3·457	3·190	2·557	3·446	3·057	1·891		
	*P* value^†^	–	0·373	0·539	0·998	0·270	0·234	0·651	0·159	0·727	0·258		
81–100 µm	FM	22·93	29·57	26·46	43·33	39·17	43·69	37·48^X^	25·69	20·81	27·93	2·210	0·136
	PP	22·93	42·45	34·86	54·13	38·84	36·09	33·36^X^	32·52	36·85	31·14	2·086	0·118
	PP + A1	22·93^ab^	15·04^b^	41·47^a^	40·41^a^	32·53^ab^	30·14^ab^	25·18^ab,X^	22·66^ab^	29·74^ab^	23·13^ab^	1·894	**0·028**
	PP + A2	22·93^ab^	39·20^ab^	55·60^ab^	56·45^a^	42·07^ab^	24·01^ab^	25·37^ab,X^	26·26^ab^	19·77^b^	21·36^ab^	2·893	**0·003**
	sem	0	4·326	4·109	3·715	3·294	2·827	1·971	2·277	2·161	2·572		
	*P* value^†^	–	0·097	0·066	0·357	0·802	0·070	**0·050**	0·521	0·004	0·553		
> 100 µm	FM	2·96^e^	4·30^e^	2·65^e^	33·22^bc^	37·59^ab^	16·08^de^	20·73^cd,XY^	48·48^a^	50·07^a^	48·67^a^	2·792	**< 0·001**
	PP	2·96^c^	7·88^c^	17·56^bc^	17·19^bc^	31·44^ab^	16·81^bc^	15·24^bc,Y^	42·47^a^	41·91^a^	50·09^a^	2·541	**< 0·001**
	PP + A1	2·96^f^	0·00^f^	7·59^ef^	31·60^cd^	37·63^bc^	20·24^de^	20·29^de,XY^	50·24^ab^	46·80^ab^	52·41^a^	2·809	**< 0·001**
	PP + A2	2·96^c^	1·68^c^	11·83^bc^	19·65^bc^	31·46^ab^	19·77^bc^	23·84^b,X^	47·70^a^	47·33^a^	49·89^a^	2·743	**< 0·001**
	sem	0	1·962	2·779	3·169	2·344	1·153	1·124	1·861	1·426	1·349		
	*P* value^†^	–	0·547	0·286	0·172	0·666	0·517	**0·040**	0·524	0·242	0·826		
Muscle fibre density (no./mm^2^)	FM	487·38^a^	420·78^a^	461·47^a^	262·91^b^	240·65^b^	236·08^b^	234·83^b,X^	184·75^b,XY^	167·96^b,XY^	164·06^b^	18·29	**< 0·001**
	PP	487·38^a^	382·97^ab^	348·99^bc^	301·75^bcd^	287·00^bcd^	234·64^cde^	214·58^de,XY^	209·95^de,X^	198·04^de,X^	152·61^e^	15·78	**< 0·001**
	PP + A1	487·38^a^	502·59^a^	386·12^ab^	277·51^bc^	244·99^c^	191·18^c^	207·57^c.XY^	159·05^c,Y^	191·06^c,X^	160·00^c^	19·69	**< 0·001**
	PP + A2	487·38^a^	412·97^ab^	348·04^abc^	297·13^bcd^	251·64^cde^	209·42^cde^	177·28^de,Y^	157·38^de,Y^	137·50^e,Y^	153·86^de^	18·58	**< 0·001**
	sem	0	22·994	20·63	7·966	9·763	8·673	6·848	6·815	7·801	4·333		
	*P* value^†^	–	0·310	0·170	0·295	0·339	0·202	**0·013**	**0·007**	**0·012**	0·794		

All values are expressed as Mean (*n* 5) and standard error of mean (sem); Mean values with different superscripts differ significantly at a 5 % probability level (*P*< 0·05). The significant *P* value (*P*<0.05) are mentioned in boldface for easy distinction from the non-significant changes.

* Significant differences (*P* value) across different sampling points (Days) for each diet are mentioned vertically at the right-most column, and mean values with different superscripts (a > b > c > d > e > f) differ significantly at a 5 % probability level (*P*< 0·05).

† Significant differences (*P* value) among dietary treatments (Diets) at each sampling point are mentioned horizontally at the bottom row for each muscle fibre size, and mean values with different superscripts (X > Y) differ significantly at a 5 % probability level (*P*< 0·05).

‡ Distribution of fibres ranging from < 20 to > 100 µm diameter is expressed as the number of specific fibre sizes present per mm^2^ area of the image at 10× magnification.

§ Experimental diets: FM, Fishmeal-based diet; PP, Plant-based diet; PP + A1, Plant–based diet supplemented with additive mixture 1 (i.e. krill meal, taurine, and organic Se); PP + A2, Plant–based diet supplemented with additive mixture 2 (i.e. L-proline, L-hydroxyproline, and vitamin C).

#### Changes in muscle fibre density and recruitment throughout the feeding trial in other diets

In corroboration with the FM diet, the PP diet-fed group showed significantly reduced muscle fibre density with respect to increasing fish size (Table 4). However, a significant reduction in muscle fibre density was observed only after Day 94 (Sampling 5) and remained significantly lower compared with Day 0 (Initial). Regarding muscle fibre recruitment pattern, the 0–20 µm diameter fibres were significantly (*P*< 0·05) higher at Day 0 than Day 15, 48, and 145–214, with no significant difference with other sampling points. However, recruitment of 0–20 µm diameter fibres was numerically higher at Day 94–115 (Sampling 5–6) as found in the FM-fed group. Moreover, the recruitment pattern of fibres ranging from 21 to 80 µm diameter was similar to muscle fibre density in the PP-fed group. Although in the PP-fed group, the recruitment of 81–100 µm diameter fibres showed no significant changes throughout the feeding trial, muscle fibres of > 100 µm diameter were found to be significantly (*P*< 0·05) higher at Day 62 (Sampling 4) and Day 145–214 (Sampling 7-Final).

In the PP + A1-fed group, a significant (*P*< 0·05) reduction in muscle fibre density with respect to Day 0 (Initial) was observed from Day 48 (Sampling 3) as the FM-fed group (Table 4). Although the recruitment pattern of 0–20 µm diameter fibres showed significant (*P*< 0·05) difference throughout the feeding trial, the *post hoc* analysis showed no difference among different sampling points (Table 4). However, Day 0–32 (Initial-Sampling 2) and Day 94–115 (Sampling 5–6) showed numerically higher recruitment of 0–20 µm diameter fibres. The recruitment pattern fibres ranging from 21 to 80 µm diameter in the PP + A1-fed group followed a similar trend of muscle fibre density in this group. However, unlike FM and PP-fed groups, the recruitment of 81–100 µm diameter fibres in the PP + A1-fed group was significantly (*P*< 0·05) higher during Day 32–48 (Sampling 2–3) than Day 15 (Sampling 1) with no difference with other sampling points. The recruitment of > 100 µm diameter fibres in the PP + A1-fed group followed a sequential order with significantly (*P*< 0·05) lower at Day 0–32 (Initial-Sampling 2), followed by Day 94–115 (Sampling 5–6) and significantly (*P*< 0·05) higher at Day 145–214 (Sampling 7-Final), followed by Day 48–62 (Sampling 3–4).

In the PP + A2-fed group, muscle fibre density and recruitment patterns of fibres ranging from < 20 to 80 µm diameter followed similar observations found in the PP + A1-fed diet (Table 4). The recruitment of 81–100 µm diameter fibres in the PP + A2-fed group showed a significantly (*P*< 0·05) higher number at Day 48 (Sampling 3) as compared with Day 180 (Sampling 8). The recruitment of > 100 µm diameter fibres in the PP + A2-fed group was significantly lower at Day 0–48 (Sampling 1–3) and Day 94–115 (Sampling 5–6) as compared with Day 145–214 (Sampling 7-Final).

#### Dietary effect on the muscle fibre density and recruitment pattern at different sampling points

A significant (*P*< 0·05) change in the muscle fibre density among different diets was observed from Day 115 to 180 (Sampling 6–8) (Table 4). A significantly (*P*< 0·05) lower muscle fibre density was found in the PP + A2-fed group than in the FM-fed group at Day 115 (Sampling 6) and in the PP and PP + A1-fed groups at Day 180 (Sampling 8). Whereas at Day 145 (Sampling 7), both additive mixture-based diets (PP + A1 and PP + A2) showed significantly (*P*< 0·05) lower muscle fibre density than the FM- and PP-fed groups.

In the muscle fibre recruitment pattern, the recruitment of 0–20 µm diameter fibres showed a significant (*P*< 0·05) difference at Day 145 (Sampling 7) among different diets with significantly (*P*< 0·05) higher numbers in the FM-fed group (Table 4). In the recruitment of 21–40 µm diameter fibres, the FM-fed group showed significantly (*P*< 0·05) higher numbers than the PP + A1-fed group at Day 32 (Sampling 2). However, the recruitment pattern of 41–60 and 61–80 µm diameter fibres showed no significant (*P*> 0·05) difference among different diets throughout the feeding trial. Although the recruitment of 81–100 µm diameter fibres showed a significant difference at *P*= 0·05 level at Day 115 (Sampling 6), the *post hoc* analysis showed no difference among the dietary groups. Finally, only the PP + A2-fed group showed a significant (*P*< 0·05) higher number of > 100 µm diameter fibres than the PP-fed group at Day 115 (Sampling 6).

### Myogenic gene expression

**Figure 3.**
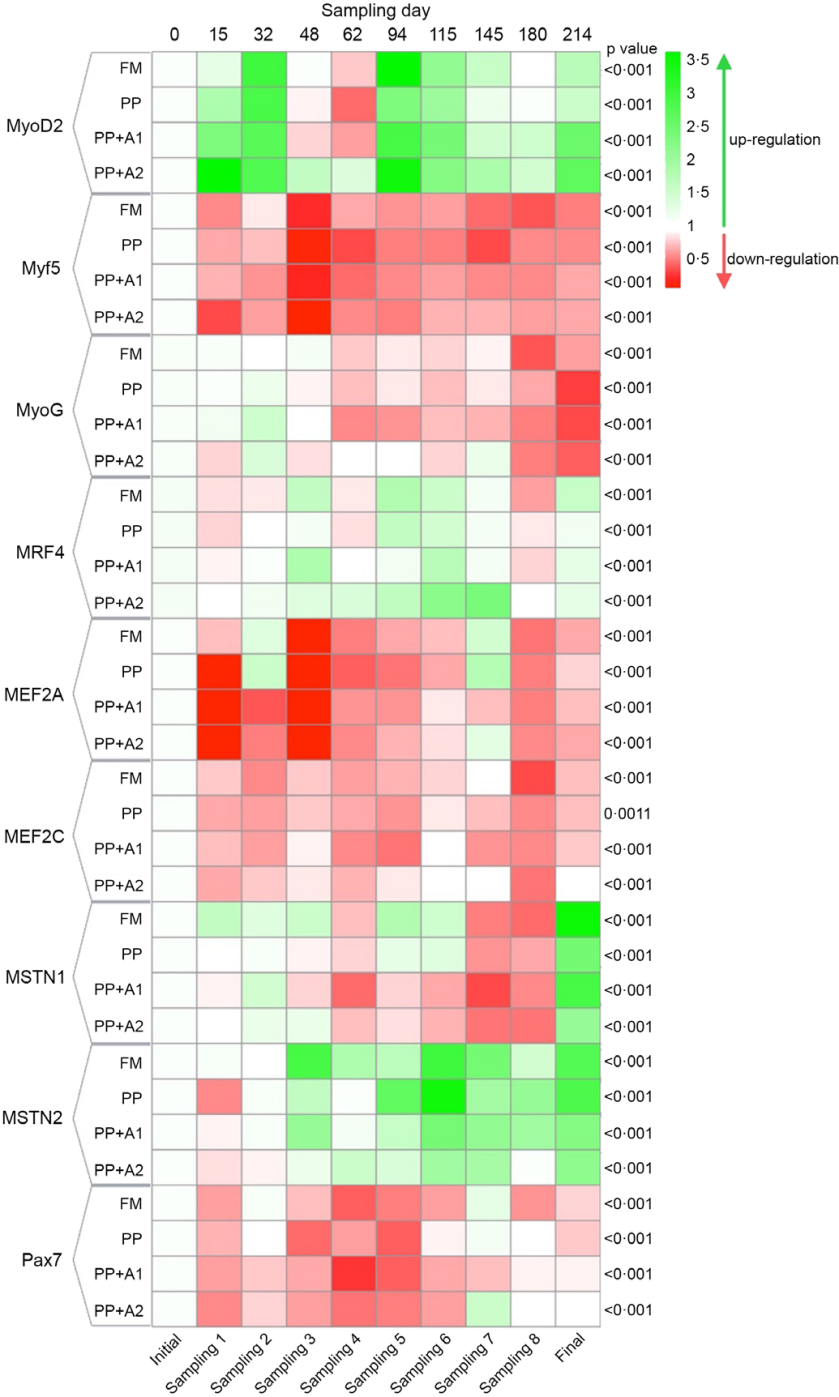
Heatmap showing expression of different myogenic genes of rainbow trout throughout seven months of feeding trial with respect to specific diets.

The expression of different myogenic genes showed a significant (*P*< 0·05) up- or downregulation when compared with respect to different sampling points (initial as control point) in rainbow trout fed different diets, as shown in Figure 3. A detailed explanation of each gene is mentioned in the following sections.

#### Expression of different myogenic regulatory factors

**Figure 4.**
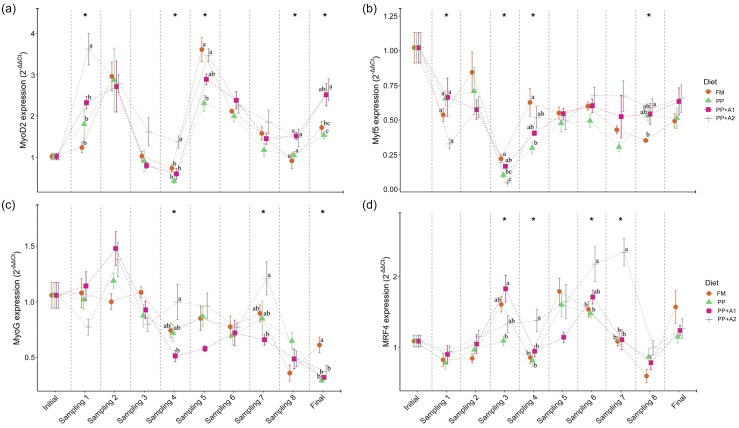
Expression of different myogenic regulatory factors (MRF), (a) Myoblast determination protein 1 homolog 2 (MyoD2), (b) Myogenic factor 5 (Myf5), (c) Myogenin (MyoG), and (d) Myogenic regulatory factor 4 (MRF4) in the muscle of rainbow trout fed four experimental diets for seven months.

The expression of all the MRF, such as MyoD2, Myf5, MyoG and MRF4, showed significant variation with respect to sampling points, irrespective of dietary treatments (Figure 3). Overall, MyoD2 showed significant upregulation at Day 15–32 (Sampling 1–2), Day 94–115 (Sampling 5–6) and finally at Day 214 (Final). However, significant downregulation was observed at Day 48–62 (Sampling 3–4) except for PP + A2 at Day 48 (Sampling 3). In terms of dietary effects, a significant upregulation of MyoD2 was found in the PP + A2 diet compared with others at Day 15 (Sampling 1) and Day 62 (Sampling 4), whereas the PP-fed group showed significant downregulation of this gene at Day 94 (Sampling 5) and Day 214 (Final) as compared with other diets (Figure 4(a)).

The Myf5 expression showed significant downregulation throughout the feeding trial in all dietary treatments compared with the initial sampling point (Figure 3). Although Myf5 expression showed significant variation among different diets at Day 15 (Sampling 1), Days 48–62 (Sampling 3–4) and Day 180 (Sampling 8), the *post hoc* analysis showed not much variation among the diets (Figure 4(b))

The MyoG expression showed significant downregulation from Day 48 (Sampling 3) to the end of the feeding trial in almost all dietary treatments, with an exception for the PP + A2-fed group at Day 145 (Sampling 7) (Figure 3). In terms of dietary treatments, the PP + A2 fed group showed significant (*P*< 0·05) high expression as compared with other diets at Day 62 (Sampling 4) and Day 145 (Sampling 7) (Figure 4(c)). Additionally, the FM-fed group showed a significant (*P*< 0·05) higher expression of MyoG at Day 214 (Final) than other diets (Figure 4(c)).

The MFR4 gene, overall, was significantly upregulated at Day 48 (Sampling 3), Day 94–115 (Sampling 5–6) and Day 214 (Final), with some variation in expression among the diets (Figure 3). In terms of dietary treatments, although the PP + A1 showed significant (*P*< 0·05) upregulation of MFR4 at Day 48 (Sampling 3) than PP, the PP + A2 showed significant upregulation of this gene at Day 62 (Sampling 4), Day 115–145 (Sampling 6–7) than other diets (Figure 4(d)).

#### Expression of myocyte enhancer factor 2 (MEF2) family

**Figure 5.**
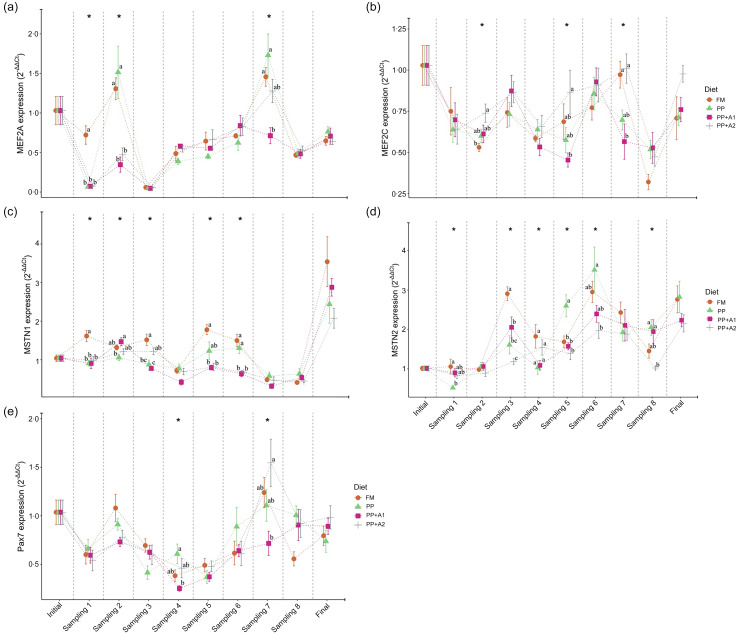
Expression of myocyte enhancer factor 2 family- (a) Myocyte enhancer factor 2A (MEF2A), and (b) Myocyte enhancer factor 2C (MEF2C); growth and differentiation inhibitors- (c) Myostatin 1 (MSTN1), and (d) Myostatin 2 (MSTN2); and satellite cell marker- (e) Paired box 7 (Pax7) in the muscle of rainbow trout fed four experimental diets for seven months.

Both MEF2 family genes, that is, MEF2A and MEF2C, showed significant downregulation throughout the feeding trial with some exceptions in the MEF2A gene for FM and PP-fed group at Day 32 (Sampling 2) and Day 145 (Sampling 7) and PP + A2-fed group at Day 145 (Sampling 7) (Figure 3). In terms of dietary effects, although the FM-fed group showed significantly (*P*< 0·05) higher expression of MEF2A at Day 15 (Sampling 1), both FM and PP showed significantly higher expression of this gene at Day 32 (Sampling 2) and Day 145 (Sampling 7) (Figure 5(a)). However, for MEF2C, the FM and PP + A2-fed group showed significantly (*P*< 0·05) higher expression of this gene at Day 94 (Sampling 5) and Day 145 (Sampling 7) (Figure 5(b)).

#### Expression of growth and differentiation inhibitors

The growth and differentiation inhibitor genes, MSTN1 and MSTN2, showed significant (*P*< 0·05) variation in their expression throughout the feeding trail, but both followed a different trend in expression (Figure 3). The MSTN1 was significantly upregulated at the end of the feeding trial (Day 214), and both the additive mixture-supplemented groups (PP + A1 and PP + A2) showed significant downregulation from Day 62 (Sampling 4) to Day 180 (Sampling 8), which is only true for FM and PP-fed groups at Day 62 (Sampling 4) and then Day 145–180 (Sampling 7–8) (Figure 3). Moreover, the dietary effect on the MSTN1 expression showed that additive mixture supplemented groups showed lower expression of this gene compared with FM and PP in most sampling points (Figure 5(c)).

Unlike MSTN1, MSTN2 showed significant upregulation from Day 48 (Sampling 3) to the end of the feeding trial in all the dietary treatments (Figure 3). Moreover, a significantly higher expression of MSTN2 was observed in the PP-fed group at Day 94–115 (Sampling 5–6) and Day 180 (Sampling 8), with the exception at Day 15 (Sampling 1), where it showed a lower expression (Figure 5(d)).

#### Expression of satellite cell marker

The satellite cell marker, Pax7, showed significant downregulation from Day 15 (Sampling 1) to Day 115 (Sampling 6) and then showed relatively higher expression at Day 145 (Sampling 7) for FM and PP + A2-fed groups but downregulated afterwards (Figure 3). In terms of dietary effects, not much variation was observed among the dietary treatments except at Day 62 (Sampling 4) when the PP-fed group showed significant (*P*< 0·05) higher expression than the PP + A1 and at Day 145 (Sampling 7) when the PP + A2-fed group showed significantly (*P*< 0·05) higher expression than PP + A1 (Figure 5(e)).

## Discussion

This study provides the first clear identification of four distinct phases of muscle growth in rainbow trout over a seven-month trial, along with insights into how additive mixture supplementation to high PP-based diets influences muscle fibre recruitment and myogenic gene expression. These findings offer context to our earlier reports evaluating growth, feed efficiency and fillet quality under different dietary regimes. In our previous report on growth and nutrient utilisation^(36)^, fish fed the high PP-based diet without additive mixtures consistently performed poorly compared with the control (FM), with lower growth and feed intake (online Supplementary Table S2). Similarly, in our fillet quality report^(37)^, the PP-fed group showed reduced textural profile and inferior fillet quality after both short- and long-term storage. In contrast, the additive mixtures-supplemented diets (PP + A1 and PP + A2) led to significant improvements over the PP diet without supplementation. Fish fed these additive mixtures not only exhibited better growth and feed efficiency^(36)^ but also demonstrated enhanced fillet texture, collagen content and post-harvest quality^(37)^. These improvements suggest that functional additive mixtures can effectively mitigate the limitations of plant-based formulations. Building on this foundation, the current study investigates the potential mechanisms of muscle development by examining muscle cellularity and myogenic gene expression patterns, as discussed in the following sections.

### Muscle histology

Although the selection of different fibre diameter ranges varies from study to study^(3,9,11,50–53)^, the common approach is to separate the muscle fibre diameter in small (0–20 μm), medium (20–100 μm) and large (> 100 μm) groups^(54)^. The presence of < 20 μm and > 100 μm diameters fibres in the myotome indicates hyperplasia and hypertrophy muscle growth in fish, respectively^(3,51–53,55)^. In this study, we have sub-grouped the medium diameter fibres further into four groups, i.e. 21–40 μm, 41–60 μm, 61–80 μm and 81–100 μm, to get a better insight into the distribution of different fibres.

We observed different diameter fibres starting from < 20 μm to > 100 μm throughout the growth period (2·22 to 350 g), irrespective of diet. The pattern of mosaic appearance depends on the distribution of different muscle fibre diameters. The high muscle fibre density indicates the recruitment of new fibres in the myotome for hyperplasia growth, as found in the 2·2 g to 9·93 g fish-fed control (FM) diet. Afterwards, the fibre density was significantly reduced, starting from 16·67 g to 355·70 g fish in control. Unlike muscle fibre density, the recruitment pattern of different diameter fibres differed at different growth stages. The presence of 0–20 µm diameter fibres at significantly higher amounts at initial (2·22 g) and Day 94 (63·69 g) concomitant with significantly lower > 100 µm diameter fibres at Day 0 to 32 (2·22–9·93 g) and Day 94–115 (63·69–88·20 g) indicates that the overall growth comes mainly from recruitment of new fibres (hyperplasia). Moreover, the recruitment pattern of fibres ranging from 21 to 80 µm diameter followed a similar trend as muscle fibre density, which inevitably confirms hyperplasia growth before Day 48 (16·67 g), but the presence of relatively higher 0–20 µm diameter fibres and lower > 100 µm diameter fibres during Day 94–115 (63·69–88·20 g) suggests hyperplasia growth in 63·69–88·20 g fish.

**Figure 6.**
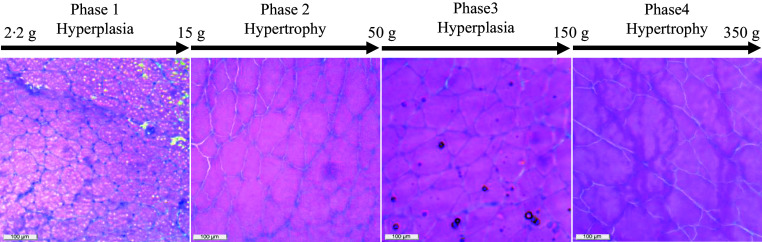
Identified growth phases based on muscle fibre recruitment pattern of rainbow trout fed the control (fishmeal-based) diet for seven months.

Based on the muscle fibre density and recruitment pattern, we have identified four distinct phases of muscle growth in rainbow trout fed control diet, such as from 2·22 to 15 g as hyperplasia, from 15 to 50 g as hypertrophy, from 50 to 150 g again as hyperplasia and finally from 150 to 350 g as hypertrophy (Figure 6). Here, we have rounded the final weight of the fish to the closest value to have a better comprehensive idea about the growth stage. Similar observations were suggested for rainbow trout with increasing levels of hyperplasia growth at 3·07–8·29 g and again at 16·3 g fish^(56)^.

The recruitment of muscle fibre in fish depends on various factors, such as species^(7)^, size and age^(8)^, nutrition^(18,20,57–59)^, strain of the same species^(55,60)^, environmental conditions such as temperature^(11)^ and photoperiod^(12)^, feeding regime^(10)^ and others. In the current study, all the other experimental diets showed a similar trend in muscle fibre density as the control (FM). However, the recruitment pattern of different fibres varied with respect to diet type. In the plant-based diet (PP), a significant reduction in muscle fibre density was observed only after the fish reached 55·32 g (Day 94) compared with 2·22 g fish (Day 0). The recruitment of fibres ranging from 21 to 80 µm diameter followed the muscle fibre density seen in the control; the recruitment pattern of 0–20 µm and > 100 µm diameter fibres was not as similar as the control group. These differences might be due to two main reasons: first, differences in diet^(18,20,57,58)^, and second, relative differences in fish body weight among these groups. Although the PP-group had a similar body length as the control, relatively lower body weight (online Supplementary Table S2) may be due to changes in muscle fibre recruitment pattern in this group^(36)^. Unlike FM- and PP-diets, both additive mixtures supplemented diets (PP + A1 and PP + A2) showed significant differences in the recruitment of 81–100 µm diameter fibres with respect to the feeding period. However, irrespective of dietary treatments, the muscle growth phases as identified in the control group (Figure 6) persist in a similar trend where the presence of fibres with diameters of < 20 µm and > 100 µm was the primary deciding factors. These observations suggest that fish length may be a better indicator for examining fibre recruitment patterns than body weight, as we observed minimal differences in length among dietary groups, except for slight variations on Day 115 and Day 214 (Table S2).

Significant changes in the muscle fibre density among different diets were seen only after Days 115 to 118, but with no difference in the final sampling. Overall, the PP + A2 showed significantly lower muscle fibre density than other diets, which may be related to the relatively lower number of < 20 µm diameter fibres with a higher number of > 100 µm diameter fibres (specifically on Day 115). This might also correlate with the higher muscle collagen content in this group^(28)^, as we reported previously on fillet quality^(37)^, along with no difference in growth in this group compared to the control^(36)^. This could explain the more relying on hypertrophic growth during this period rather than recruiting new fibre through a large fibre-splitting process^(53)^. Overall, the FM diet group, which consistently showed more small- and medium-sized muscle fibres than other dietary groups, suggests that FM promotes a more active muscle fibre recruitment process, potentially leading to faster overall muscle growth^(36)^. Although plant-based protein did not change the overall muscle cellularity, a similar observation was reported in rainbow trout fed two types of plant-based proteins^(20)^. Whereas the same author suggested a change in the medium diameter fibres when fishmeal was substituted at 75–100 % level in the diet of rainbow trout^(18)^.

### Myogenic gene expression

MRF are critical transcription factors in initiating and regulating muscle development^(1,4)^. The expression of four MRF investigated in this study, namely MyoD2, Myf5, MyoG and MRF4, showed dynamic changes throughout the feeding trial, suggesting modulation by both dietary and life-stage factors^(15,18,20)^. The wave-like pattern of MyoD2 expression with upregulation at specific time points (Day 15–32, Day 94–115 and Day 214, which corresponds to 5–10 g, 50–75 g and 350 g, respectively) and downregulation at others (Day 48–62, which corresponds to 15–20 g) suggests a pulsatile activation of MyoD2 during muscle growth, likely reflecting the phases of myoblast proliferation and differentiation^(10,15)^. The PP + A2 diet notably promoted MyoD2 upregulation at certain points. Supplementation of hydroxyproline and vitamin C, part of additive mixture 2 in PP + A2, supported higher expression of the MyoD2 gene in triploid crucian carp (*Carassius auratus*)^(28)^ and pacu (*Piaractus mesopotamicus*)^(27)^, respectively. Myf5 was mostly downregulated, while MyoG expression declined after Day 48, except for the PP + A2 group at Day 145. MyoG is a key marker of terminal muscle differentiation^(10,15)^, showing downregulation over time, indicating a shift towards mature muscle fibres. The PP + A2 diet sustained MyoG expression at Day 145, potentially indicating extended myofibre growth in this group^(29,61)^. MRF4 expression displayed a pattern with upregulation at Day 48, Day 94–115 and Day 214. MRF4 is known to be involved in both muscle proliferation and differentiation^(10,29)^. Dietary changes showed additive mixtures supplemented diets (PP + A1, and PP + A2) promoted MRF4 upregulation at specific points. Johansen and Overturf (2005) observed peaks in MyoD2 expression during the swim-up fry stage and Myf5 expression at the 25-g stage in rainbow trout^(15)^, suggesting two waves of hyperplasia along with upregulation of these genes in spawning rainbow trout. Here, MyoD2 expression peaks suggest that the hyperplasia event mostly corresponds to rainbow trout from 2·2 g to 30 g and again 50 to 75 g fish. However, MyoD2 upregulation in 350 g fish may reflect maturation instead of hyperplasia growth as found in the spawning rainbow trout^(15)^. Similar expression patterns of MyoD2 and MRF4 suggest their key role in muscle proliferation and growth through hyperplasia in this study.

MEF2A and MEF2C displayed a general downregulation throughout the experiment, with some exceptions. This might indicate a lesser role for these factors in hypertrophy compared with the findings of Johansen and Overturf (2005), who observed peak expression of MEF2A and MEF2C during the swim-up fry stage, associating them with early muscle hypertrophy and a second increase in MEF2A expression at the 25-g stage, potentially contributing to renewed hypertrophy^(15)^. However, brief periods of upregulation in some dietary groups (FM, PP, PP + A2) in this study suggest potential dietary modulation of MEF2A and MEF2C activity.

Myostatin, also known as growth differentiation factor 8 (GDF-8), is present in multiple genotypes in fish with unique temporal and spatial expression patterns, suggesting potential roles in regulating the growth and development of other tissues, like the brain and gonads, in addition to muscle^(16)^. The significant upregulation of MSTN1 with the progress of the feeding trial (Day 214) suggests a potential inhibitory role on muscle growth in the later stages. However, the lower MSTN1 expression in the additive mixture-supplemented groups (PP + A1 and PP + A2) compared with FM and PP implies that the additive mixture might counteract MSTN1-mediated growth inhibition, potentially contributing to enhanced growth as observed in the additive mixture-supplemented groups compared to others, specifically PP^(36)^. Unlike MSTN1, MSTN2 expression exhibited a continuous upregulation throughout the experiment, suggesting a possible cumulative inhibitory effect of MSTN2 on muscle growth. Moreover, the PP diet showed a significantly higher expression of MSTN2 at certain points (Day 94–115, Day 180), suggesting a potentially stronger inhibitory influence of plant-based protein on growth, which can be alleviated through addictive mixtures supplementation found for PP + A1 and PP + A2 groups^(36)^. A similar pattern of MSTN expression was reported by Johansen and Overturf (2005), who suggested myostatin expression might increase in response to muscle growth^(15)^. While both isoforms of MSTN act as negative muscle growth regulators, the current study suggests their expression patterns and responsiveness to diet can differ.

Satellite cells are a population of muscle stem cells crucial for muscle growth and repair^(17)^. The Pax7 gene is a marker for muscle satellite cells and is essential for muscle development during growth and repair after injury in fish^(17)^. Pax7 displayed a downregulation trend from Day 15 to Day 115 (5–75 g), suggesting a potential decrease in the satellite cell pool as the feeding trial progressed. However, a slight upregulation was observed at Day 145 (150 g) for the FM and PP + A2 groups, followed by another downregulation. This might indicate transient activation of satellite cells for muscle growth spurts in these groups. However, minimal variation in Pax7 expression was observed among diets, except for a few specific points, suggesting dietary factors might have a limited influence on the overall satellite cell pool in this experiment^(10)^.

### Correlating muscle histology with myogenic gene expression

The hyperplasia phases identified (2·2–15 g, 50–150 g) aligned with upregulation of MyoD2, a key regulator of myoblast proliferation and differentiation^(10,15)^. Conversely, the hypertrophy phases (15–50 g, > 150 g) showed MyoD2 downregulation, indicating a shift towards muscle fibre growth over new fibre formation^(10,15)^. MyoG upregulation during certain periods (e.g. PP + A2 at Day 145) coincides with more large-diameter muscle fibres (> 100 µm), indicating terminal differentiation and hypertrophy. These hypertrophy phases also coincide with lower MEF2A and MEF2C expression. Unlike earlier studies^(15)^, this suggests MEF2 may have played a lesser role in regulating hypertrophic growth in this study. Among the MRF, MyoD2 appears particularly crucial in promoting muscle fibre recruitment and hyperplasia in rainbow trout. Muscle fibre recruitment differed between FM and PP diets, likely due to differences in nutrient availability as reported in our previous report with relatively lower feed intake in the PP diet^(36)^. The additive mixtures (PP + A1 and PP + A2) modulated MyoD2, MyoG and MRF4 expressions, suggesting their potential to influence muscle growth dynamics, as seen in growth, nutrient utilisation and fillet quality^(36,37)^. The PP + A2 diet promoted muscle hypertrophy, with more large-diameter fibres (> 100 μm) and lower muscle fibre density at mature stages. MSTN1 and MSTN2 upregulation in later stages coincided with reduced muscle fibre density and recruitment, indicating muscle growth inhibition. Lower MSTN1 expression in PP + A1 and PP + A2 diets potentially enhances muscle growth by counteracting this inhibition. The downregulation of Pax7 from Day 15 to Day 115 (5–75 g) suggests a decrease in the satellite cell pool, with minimal dietary impact.

### Conclusions

Muscle histology revealed four growth phases: hyperplasia (2·2 g to 15 g), hypertrophy (15 g to 50 g), hyperplasia (50 g to 150 g) and hypertrophy (150 g to 350 g). Correlating muscle histology with myogenic gene expression provided insight into how hyperplasia, hypertrophy and dietary factors interact to regulate muscle growth in rainbow trout. The findings suggest that MyoD2 and MRF4 are key drivers of hyperplasia, while myostatin isoforms act as negative growth regulators. The additive mixtures further modulate these processes, potentially influencing muscle growth strategies via gene expression. Overall, these identified growth phases could help optimise dietary inclusion by providing a specific diet composition tailored for each growth phase, supporting precision nutrition in aquaculture.

## Supporting information

Singha et al. supplementary material Singha et al. supplementary material

## Supplementary material

For supplementary material/s referred to in this article, please visit https://doi.org/10.1017/S0007114525105175
