# Supplementary material for: Muscle hyperplasia and hypertrophy dynamics in rainbow trout (Oncorhynchus mykiss): impact of high plant-based protein and additive mixtures on muscle physiology
Source: Br J Nutr. 2025 Sep 23;134(8):617–33. doi: 10.1017/S0007114525105175 (PMC12722011; doi:10.1017/S0007114525105175)
Supplement: Singha et al. supplementary material [file S0007114525105175sup001.pdf]

Supplementary file

Table S1. Water quality parameters of the recirculating aquaculture system (RAS) during the seven-month feeding trial.

Parameters	Sampling 1	Sampling 2	Sampling 3	Sampling 4	Sampling 5	Sampling 6	Sampling 7	Sampling 8	Sampling Final
	Day 15	Day 32	Day 48	Day 62	Day 94	Day 115	Day 145	Day 180	Day 214
Temperature (ºC)	16.60	13.40	13.90	14.20	14.30	15.60	16.20	15.30	15.40
Dissolved O_2 (mg/L)	6.04	5.97	5.85	5.13	5.01	5.69	5.61	5.98	5.20
pH	7.43	7.42	7.59	7.58	7.64	7.50	7.60	7.63	7.47
Ammonia-N (mg/L)	0.02	<0.01	<0.01	0.03	0.01	0.01	<0.01	0.01	<0.01
Nitrite-N (mg/L)	0.15	0.10	0.10	0.11	0.10	0.18	0.10	0.12	0.10

Table S2. Performance indices of rainbow trout fed four experimental diets for seven months.

Performance indices[1]	Diets[2]	Sampling 1	Sampling 2	Sampling 3	Sampling 4	Sampling 5	Sampling 6	Sampling 7	Sampling 8	Sampling Final
		Day 15	Day 32	Day 48	Day 62	Day 94	Day 115	Day 145	Day 180	Day 214
Final weight (g)	FM	4.97[a]	9.93[a]	16.67[a]	22.59[a]	63.69[a]	88.20[a]	144.27[a]	221.76[a]	355.70[a]
	PP	4.68[b]	9.01[b]	14.69[b]	20.19[b]	55.32[b]	79.91[b]	135.50[b]	203.88[b]	320.62[b]
	PP+A1	4.81[ab]	9.44[ab]	15.79[ab]	21.79[ab]	58.68[ab]	84.96[ab]	145.68[ab]	223.91[a]	362.76[a]
	PP+A2	4.66[b]	9.03[b]	15.04[b]	21.00[ab]	57.32[b]	83.04[ab]	139.41[ab]	220.74[a]	359.34[a]
	SEM	0.035	0.111	0.23	0.335	0.95	0.957	1.424	2.109	4.348
	p value	**<0.001**	**0.002**	**0.003**	**0.053**	**0.004**	**0.007**	**0.029**	**<0.001**	**<0.001**
Length[3] (cm)	FM	7.41	8.68	10.13	11.52	15.66	18.55[ab]	21.78	25.44	29.52[a]
	PP	7.27	8.68	9.9	11.39	15.61	17.74[b]	21.64	25.24	28.12[b]
	PP+A1	7.22	8.58	10.03	11.76	15.92	18.18[ab]	22.13	25.32	29.51[a]
	PP+A2	7.3	8.69	10.07	11.33	15.51	18.84[a]	21.71	25.12	29.47[a]
	SEM	0.094	0.119	0.125	0.211	0.259	0.228	0.228	0.196	0.306
	p value	0.58	0.89	0.625	0.513	0.727	**0.02**	0.468	0.704	**0.012**
Fulton's condition factor (K)	FM	1.25	1.32	1.33	1.23	1.47	1.39[a]	1.4	1.38	1.39
	PP	1.3	1.31	1.26	1.2	1.42	1.35[ab]	1.34	1.36	1.44
	PP+A1	1.3	1.27	1.27	1.21	1.42	1.31[b]	1.37	1.34	1.43
	PP+A2	1.25	1.27	1.24	1.17	1.4	1.32[b]	1.38	1.36	1.45
	SEM	0.014	0.013	0.014	0.011	0.012	0.01	0.012	0.009	0.012
	p value	0.479	0.273	0.113	0.275	0.222	**0.022**	0.269	0.684	0.201
Daily feed intake ($g.fish^{-1}.day^{-1}$)	FM	0.14	0.24	0.4	0.38	1.12	1.06	1.26[c]	1.53[c]	3.24[ab]
	PP	0.14	0.22	0.36	0.34	1.05	1.07	1.38[b]	1.48[c]	2.66[c]
	PP+A1	0.14	0.23	0.39	0.38	1.04	1.14	1.57[a]	1.72[a]	3.41[a]
	PP+A2	0.13	0.22	0.36	0.37	1.06	1.09	1.44[b]	1.63[b]	3.14[b]

	**SEM**	0.003	0.004	0.008	0.008	0.012	0.013	0.027	0.022	0.068
	**p value**	0.718	0.087	0.186	0.275	0.105	0.154	**<0.001**	**<0.001**	**<0.001**
**Feed conversion ratio (FCR)**	**FM**	0.70	0.83	0.95^{b}	0.90	0.87^{b}	0.91	0.68^{b}	0.78	0.87
	**PP**	0.81	0.86	1.02^{a}	0.87	0.95^{a}	0.92	0.77^{ab}	0.78	0.84
	**PP+A1**	0.78	0.83	0.97^{ab}	0.89	0.91^{ab}	0.91	0.78^{a}	0.83	0.87
	**PP+A2**	0.77	0.85	0.97^{ab}	0.87	0.94^{a}	0.9	0.75^{ab}	0.78	0.83
	**SEM**	0.02	0.009	0.009	0.011	0.009	0.016	0.013	0.011	0.011
	**p value**	0.29	0.467	**0.048**	0.75	**0.003**	0.967	**0.011**	0.364	0.379

All values are expressed as Mean (n = 5) and standard error of mean (SEM); Mean values with different superscripts differ significantly at a 5% probability level ($p < 0.05$).

[1]Performance indices except ‘Length’ were sourced from the ‘Supplementary file’ in Singha et al. (2025) [(36)].

[2]Experimental diets: FM, Fishmeal-based diet; PP, Plant-based diet; PP+A1, Plant-based diet supplemented with additive mixture 1 (i.e., krill meal, taurine, and organic Se); PP+A2, Plant-based diet supplemented with additive mixture 2 (i.e., L-proline, L-hydroxyproline, and vitamin C).

[3]Average length of six randomly selected fish represents the length of each replication (tank) for different diets
